# Role of MCP-1/CCR2 axis in renal fibrosis: Mechanisms and therapeutic targeting

**DOI:** 10.1097/MD.0000000000035613

**Published:** 2023-10-20

**Authors:** Shiyang He, Lan Yao, Jun Li

**Affiliations:** a The Fifth Affiliated Hospital of Zunyi Medical University, Zhuhai, China; b Basic and Applied Laboratory of Traditional Chinese Medicine, Zunyi Medical University Zhuhai Campus, Zhuhai, China; c Blood Purification Center, the First Affiliated Hospital of Zhengzhou University, Zhengzhou, China.

**Keywords:** chemokine, inflammation, macrophages, MCP-1/CCR2 axis, renal fibrosis

## Abstract

Renal fibrosis is a common pathological manifestation in various chronic kidney diseases. Inflammation plays a central role in renal fibrosis development. Owing to their significant participation in inflammation and autoimmunity, chemokines have always been the hot spot and focus of scientific research and clinical intervention. Among the chemokines, monocyte chemoattractant protein-1 (MCP-1), also known as C-C motif chemokine ligand 2, together with its main receptor C–C chemokine receptor type 2 (CCR2) are important chemokines in renal fibrosis. The MCP-1/CCR2 axis is activated when MCP-1 binds to CCR2. Activation of MCP-1/CCR2 axis can induce chemotaxis and activation of inflammatory cells, and initiate a series of signaling cascades in renal fibrosis. It mediates and promotes renal fibrosis by recruiting monocyte, promoting the activation and transdifferentiation of macrophages. This review summarizes the complex physical processes of MCP-1/CCR2 axis in renal fibrosis and addresses its general mechanism in renal fibrosis by using specific examples, together with the progress of targeting MCP-1/CCR2 in renal fibrosis with a view to providing a new direction for renal fibrosis treatment.

## 1. Introduction

Renal fibrosis is a general pathological manifestation of various chronic kidney diseases (CKD) that develop into end-stage renal disease (ESRD).^[1,2]^ A global chronic kidney disease research report published in The Lancet in 2020 showed that there were approximately 700 million CKD patients worldwide, accounting for approximately 9.1% of the world population. In China it accounts for the largest number of CKD patients, approximately 132 million,^[3]^ which has been a heavy burden on social and economic development. Kidney repair is a complex pathological process that includes not only the participation of various intrinsic renal cells and renal blood vessels, but also a variety of immune cells and inflammatory factors such as chemokines. Pathological repair of the renal parenchyma and/or interstitium after kidney damage leads to renal fibrosis. Inflammation plays a crucial role in renal fibrosis. Chemokines in the inflammatory response have been a popular research topic for scholars. Among them, monocyte chemoattractant protein-1 (MCP-1) and C–C chemokine receptor type 2 (CCR2), the main receptors of MCP-1, are widely studied chemokines in renal fibrosis. MCP-1 is a critical chemokine which regulates the biological activities of monocytes/macrophages. It can chemotivate and recruit a variety of inflammatory cells to damaged sites.^[4,5]^ CCR2 is the main but not the only receptor for MCP-1.^[6]^ The MCP-1/CCR2 axis activate monocytes and macrophages, stimulating the release of interleukin-1 (IL-1), IL-6, and tumor necrosis factor-α (TNF-α).^[7]^ Conversely, macrophages activated by the MCP-1/CCR2 axis also secrete IL-10, which promote tissue repair.^[2,7]^ MCP-1 is related to inflammatory diseases and neurodegenerative diseases, such as atherosclerosis, rheumatoid arthritis, and neurodegenerative diseases.^[7]^ It is a promising therapeutic target for inflammatory and neurodegenerative diseases. The basic principle of targeting MCP-1/CCR2 to treat related diseases in human beings is to use drugs to block MCP-1 or CCR2, inhibit the activation and conduction of MCP-1/CCR2 axis, thereby reducing the inflammatory cells and pro-inflammatory cytokines.^[8,9]^ In addition, the MCP-1/CCR2 axis is also a very important participant in chemokine signaling in renal fibrosis. More and more scholars show great interest in the MCP-1/CCR2 axis because of its significant participation in renal fibrosis. Here, we focus on the complex physical processes and general mechanisms of MCP-1/CCR2 axis in renal fibrosis in this review. In particular we describe the MCP-1/CCR2 axis as a potential target for treating renal fibrosis by using specific examples.

## 2. The structure and function of MCP-1 and CCR2

Chemokines, with low molecular weights, are a series of highly conserved secreted proteins that transduce signals through G protein-coupled receptors, and participate in diverse biological processes such as chemotaxis, angiogenesis, and hematopoiesis. Currently, more than 50 kinds of chemokines have been identified, and their molecular weights are very small, between 8 and 12 kDa.^[10]^ They all contain 4 highly conserved cysteine residues. The first 2 cysteine residues are near the N-terminus of the mature protein, the third is at the center of the molecule, the fourth is near the C-terminus. There is a loop of approximately 10 amino acid residues after the first 2 cysteine residues, called the N-loop, 4 conserved cysteine residues which guarantee the tertiary structure of the chemokine. Chemokines are split into 4 main subfamilies according to the number of the cysteines residues and the arrangement of the 2 conserved cysteine residues close to the N-terminus: C, CC, CXC, and CX3C.^[10–12]^ The C chemokine lacks the first and third cysteine residues. At the N-terminus of CC chemokines, 2 cysteine residues are juxtaposed directly. In CXC chemokines, a single amino acid is located between the first 2 cysteines, and there are 3 amino acids in the CX3C subfamily separating the first 2 cysteine residues. The CC chemokine family consists of more than 20 members (CCL1-28), which mainly undergo chemotaxis and activate monocytes and certain T cell subsets, DC cells, B cells, and NK cells. MCP-1, also called C-C motif chemokine ligand 2, was the first identified and most deeply studied chemokine in the CC chemokine subfamily.

### 2.1. Structure and function of MCP-1

The MCP-1 gene is situated in human chromosome 17q11.2-q21.1.^[13]^ Mature human MCP-1 is a small protein (13 kDa) comprising 76 amino acid residues.^[14]^ The primary structure of biological activity has 2 important regions: amino acids 10 to 13 and amino acids 34 to 35. The mutation of the former reduces biological activity, while the latter leads to a complete loss of activity of MCP-1.^[6]^ Positions 11, 12, 36, and 52 of the MCP-1 protein molecule are the positions of 4 conserved cysteine residues. Disulfide bonds exist between them, and the disulfide bonds formed by Cys11-Cys36 and Cys12-Cys52 form a left-handed helix. Thus, disulfide bonds may be necessary for MCP-1 biological activity. The secondary structure of MCP-1 is formed by a 4-stranded β-sheet, a short unstructured N-terminal loop, and a C-terminal α-helix, which lies above the Greek bond formed by β-folding.^[15,16]^ The N-terminal plays an important role in receptor activation. Furthermore, missing residues at the N-terminal lead to activity loss. Unlike humans, mouse MCP-1 is a protein with 125 amino acids, which has a high sequence similarity with human MCP-1 at the N-terminus. The biggest difference from human homologs is that there are 49 residues at the C-terminal of mouse MCP-1.^[17,18]^

In general, MCP-1 exists as a monomer or a dimer. MCP-1 monomers combine with each other at the N-terminus to form dimers, whereas the 2 C-terminal α-helices enclose the chamber. It has been speculated that there is a receptor-binding functional area in this domain that can bind to receptors. The N-terminus is a chemotactic functional region that can activate receptors to mediate downstream signaling. Most researchers believe that both the monomeric and dimeric forms of MCP-1 are biologically active.^[19]^

In human MCP-1, there is an N-linked glycosylation site (position 14 is a potential N-linked glycosylation site) and multiple O-linked glycosylation sites, which are more abundant of murine MCP-1 than those of human MCP-1. Glycosylation not only changes the spatial conformation and biological activity of chemokines, but also plays a significant role in molecular recognition and cell signaling. The glycosylation reduces the chemotactic activity of MCP-1.^[6]^ Accordingly, it is speculated that the glycosylation site of MCP-1 may be a promising target for drug therapy.

MCP-1 can be produced by multiple cells, such as monocytes/macrophages, smooth muscles, mesangial cells, endothelial cells, fibroblasts, and microglial cells.^[10]^ Monocytes/macrophages are the main sources of its production.^[17]^ Multiple pro-inflammatory factors can upregulate the expression of MCP-1, IL-1β, TNF-α, and transforming growth factor-β (TGF-β).^[20]^ MCP-1 expression is highly induced by inflammation and nuclear factor kappa-B (NF-κB) activation.^[21,22]^ Besides, oxidative stress also upregulated the expression of MCP-1.^[23,24]^ While p38 inhibitor repressed MCP-1 mediated Chemotaxis.^[6]^ When MCP-1 binds to CCR2, it mediates signal transduction, and monocyte chemokine-1-inducing protein-1 is a direct downstream target. Monocyte chemokine-1-inducing protein-1, which regulates the expression of MCP-1, IL-1β and TNF-α, is a transcriptional activator.^[25]^ The expression of MCP-1 is upregulated when human and mouse renal mesangial cells are stimulated by various injury-related factors.

### 2.2. Structure and function of CCR2

**Figure 1.**
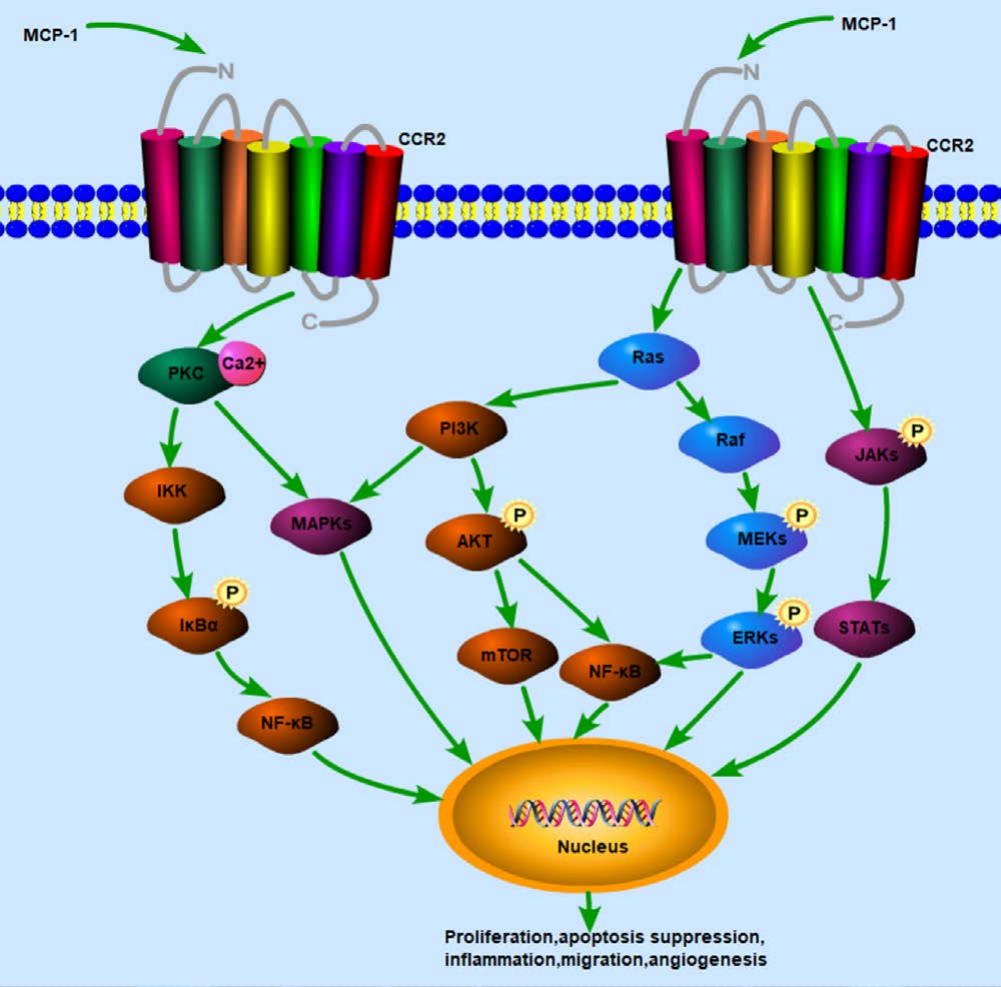
Related signaling pathways of the MCP-1-CCR2 axis. The MCP-1/CCR2 axis is activated after MCP-1 binds to CCR2. Then a series of downstream signals, including JAK/STATs, PI3K/MAPKs, PI3K/Akt, Ras/Raf-1/MEKs/ERKs and nuclear factor kappa-B (NF-κB) are activated.^[17]^ And activation of those pathways modulate a variety of transcription factors and genes, which promote the progress of renal fibrosis. Akt = protein kinase B (PKB), CCR2 = C–C chemokine receptor type 2, ERK = extracellular signal-regulated kinase, JAK = janus kinase, MAPKs = mitogen-activated protein kinase, MCP-1 = monocyte chemoattractant protein-1, MEK = mitogen-activated protein kinase kinase, PI3K = phosphatidylinositol 3 kinase, STAT = signal transducer and activator of transcription.

CCR2 has 2 subtypes (CCR2A and CCR2B).^[26]^ CCR2B is mainly localized on the cell surface, whereas CCR2A is mainly localized in the cytoplasm.^[26,27]^ Interestingly, chemokines and their receptors show significant redundancy. A ligand can bind to several receptors; conversely, a receptor can bind to several ligands as well.^[13,28]^ CCR2 can bind to C-C motif chemokine ligand 2 (MCP-1), CCL7, CCL8, CCL12, CCL13, and CCL16.^[26]^ Interestingly, CCR2 is considered a possible receptor for Cytl1.^[29]^ And Cytl1 is structurally similar to MCP-1 and chemotaxis on monocyte and macrophages via receptor CCR2B-ERK pathway. Both Cytl1 and MCP-1 can promote extracellular signal-regulated kinase (ERK) phosphorylation, which is essential for its chemotactic activity in human monocyte.^[29]^ And NF-κB may be the downstream target of ERK (Fig. 1). Cytl1/CCR2 may mediate ERK signaling pathway and participate in the pathogenesis of rheumatoid arthritis and osteoarthritis, and may be related to many other diseases, including cardiac fibrosis, neuroblastoma, heart failure, and smoking-associated health impairment. Cytl1 may also mediate the TGF-β-Smad signal pathway,^[30]^ which plays a crucial role in inflammation and renal fibrosis.^[31]^ MCP-1 exhibits the highest activity in ligands that bind to CCR2.^[26]^ The combination of CCR2 and MCP-1 was most effective in transducing downstream signaling.^[25]^ CCR2 consists of a C-terminus, 3 intracellular and 3 extracellular hydrophilic loops, 7 helical transmembrane domains, and an acidic N-terminus. N-terminal domains determine the specificity of ligand binding, whereas G proteins coupled to the C-terminus of CCR2 mediate intracellular signal conduction after activation of the receptor, causing target cell effects.^[26]^

CCR2 is present on multiple cellular populations, including monocytes, macrophages, endothelial, dendritic, and T cells, and transduces extracellular signals by interacting with MCP-1.^[4,5,26]^

### 2.3. MCP-1/CCR2 axis

After MCP-1 binds to CCR2, it activates MCP-1/CCR2 axis and plays a role in the pathogenesis of various diseases.^[4,32–34]^ The Chemotaxis of MCP-1/CCR2 axis to monocytes/macrophages is dominant in the CC family. The increase in MCP-1 levels is related to glomerular macrophage infiltration in many diabetic nephropathy (DN) models.^[35]^ High levels of MCP-1 can be used to identify renal fibrosis and predict adverse renal outcomes in the future.^[36]^ MCP-1 and CCR2 are significantly increased in damaged kidneys.^[37–40]^ After kidney injury, MCP-1 is highly expressed, recruiting and activating monocyte-macrophages to the injury site, and specifically binds to the receptor CCR2 on monocyte-macrophages and exerts biological effects through the calcium-dependent protein kinase C-mediated signaling pathway. Inhibition of MCP-1/CCR2 axis can reduce the infiltration of macrophages into kidneys, thereby reducing inflammation and renal fibrosis in the damaged kidneys.^[40]^ When MCP-1 binds to CCR2, a series of downstream signaling cascades, including janus kinase/signal transducer and activator of transcriptions, PI3K/mitogen-activated protein kinase, NF-κB, Ras/Raf-1/MEK/ERK and PI3K/Akt signaling pathways, can be activated (Fig. 1).^[20,41]^ The activation of those pathways can modulate a variety of transcription factors and genes, and play critical roles in renal fibrosis.

## 3. Mechanism of MCP-1/CCR2 axis in renal fibrosis

### 3.1. MCP-1/CCR2 axis promotes renal fibrosis by recruiting inflammatory cells

**Figure 2.**
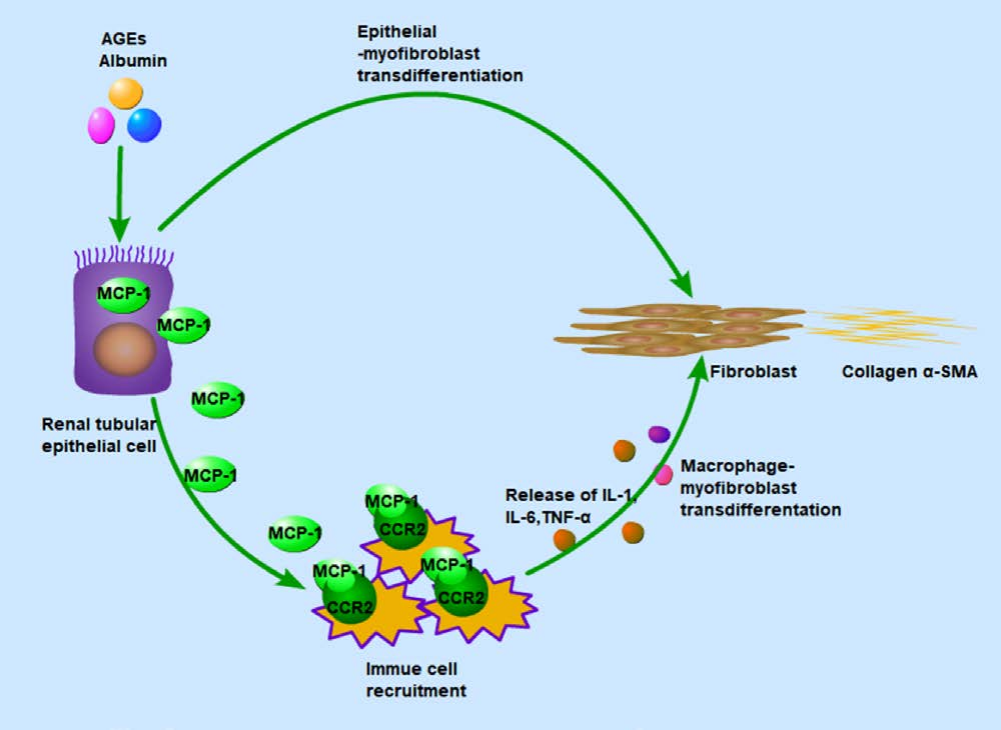
The MCP-1-CCR2 axis promotes renal fibrosis progression by recruiting inflammatory cells. MCP-1 generated from tubular epithelial cells prompts the recruitment of CCR2-positive immue cells to the site of damaged kidney after binding to CCR2 after kidney injury.^[2]^ MCP-1 promotes the progression of kidney inflammation and fibrosis. CCR2 = C–C chemokine receptor type 2. MCP-1 = monocyte chemoattractant protein-1.

Inflammation is a critical factor that drives renal fibrosis, and continuous inflammatory stimulation is an important pathogenesis of CKD.^[42]^ Numerous reports demonstrated that MCP-1/CCR2 axis plays a vital role in macrophage chemotaxis during both the occurrence and development of inflammation.^[43]^ The MCP-1/CCR2 axis are related to inflammation and progression of kidney fibrosis. CCR2 is released from macrophages, dendritic cells, and T cells. MCP-1 is released after kidney injury, prompting the recruitment of CCR2-positive monocytes/macrophages, dendritic cells, and fibroblasts to the injury site after binding to CCR2 and promotes the progression of kidney inflammation and fibrosis (Fig. 2).^[2,38,44–46]^ The MCP-1/CCR2 axis does not directly mediate polarization of pro-fibrotic macrophages. Its main role during the transition from monocytes to pro-fibrotic macrophages is to induce cell homing and persistent pro-fibrotic macrophages, while breast regression protein 39, expressing by kidney cells, can directly promote the activation of pro-fibrotic macrophages.^[45]^ Those suggest that after kidney damage, the MCP-1/CCR2 axis promotes renal inflammation and fibrosis by recruiting monocytes/macrophages, and dendritic cells to the damaged site.

### 3.2. MCP-1/CCR2 axis induces transdifferentiation of macrophages into myofibroblasts

Extracellular matrix (ECM) mainly produced by myofibroblasts during tissue fibrosis. In renal fibrosis, pericytes, endothelial cells, and tubular epithelial cells can all be transdifferentiated into myofibroblasts,^[47]^ which increases the production of ECM. Recent studies have shown that activated macrophages can not only be recruited to the damaged kidney to promote the renal inflammatory response but also transdifferentiate into myofibroblasts. Wu et al showed that Twist1 plays a critical role in macrophage-myofibroblast transdifferentiation (MMT) in unilateral ureteral obstruction (UUO) model kidneys, and MCP-1/CCR2 axis is likely to be one of the main pathways leading to Twist1-mediated UUO renal fibrosis.^[39]^ A biopsy study showed that patients with active fibrotic nephropathy, CD68+αSMA + cells, and intermediate MMT, were found to have active fibrotic nephropathy. The cell number correlates with the total number of myofibroblasts in active fibroblast tissues. In addition, most CD68+αSMA + cells co-expressed CD206, indicating that the cells involved in MMT were mainly M2 macrophages.^[48]^ MCP-1/CCR2 axis is a key signal axis for monocyte chemotaxis and macrophage activation. Therefore, MCP-1/CCR2 axis may be a necessary signaling pathway for macrophage activation and transdifferentiation into myofibroblasts.

## 4. MCP-1/CCR2 axis and various renal fibrotic diseases

MCP-1 and its receptor CCR2 are considered key mediators of kidney fibrosis, and their expression is increased in various CKD, such as adriamycin nephropathy, DN, rapidly progressive glomerulonephritis, lupus nephritis.^[49,50]^ MCP-1/CCR2 axis plays a vital role in DN, autoimmune kidney disease, and obstructive nephropathy.

### 4.1. MCP-1/CCR2 axis and DN

Fibrosis is one of the primary pathological changes associated with DN. At present, DN is the primary cause of ESRD. Patients with DN have a 3 to 12 times higher mortality rate than those with simple diabetes mellitus.^[51]^ MCP-1 is not only a marker of early DN, but also a key mediator of DN.^[52]^ An et al showed that MCP-1 stimulated and recruited monocytes/macrophages to damaged kidneys in DN. The increase in MCP-1 could aggravate the release of oxidoreductase and apoptosis of membrane podocytes, leading to urinary protein leakage.^[53]^ MCP-1 not only affects the activation of inflammatory signaling pathways but also promotes urinary protein leakage. In a case-cohort study, 894 patients with DN were observed for approximately 8.7 years, showing that higher levels of plasma MCP-1 were related to DN progression.^[54]^ In patients with DN, MCP-1 is an independent risk factor for ESRD and has important clinical value for evaluating DN prognosis. As previously mentioned, MCP-1 participates in progressive renal fibrosis by binding to its receptor, CCR2.^[2]^ This indicates that MCP-1/CCR2 axis seems to be a promising target for treating DN.

### 4.2. MCP-1/CCR2 axis and autoimmune kidney disease

Renal fibrosis is an important process in various autoimmune nephropathies, such as lupus nephritis, focal segmental glomerulosclerosis, and IgA nephropathy. Lupus nephritis is a common autoimmune kidney disease. MCP-1, a marker of lupus nephritis,^[34,55]^ has received much attention in lupus nephritis because the MCP-1/CCR2 axis mediates the recruitment and infiltration of macrophages in injured kidneys.^[56,57]^ In human and mouse lupus nephritis, macrophage infiltration is associated with disease progression and poor prognosis. The MCP-1/CCR2 axis promotes the infiltration of glomerular macrophages and an increase in intracapillary cells in lupus nephritis. In addition, MCP-1 promotes renal macrophage infiltration, which may also be related to the activation of the signal transducer and activator of transcription 3 signaling pathway by IL-22.^[56]^ A prospective cohort study which included 100 patients with autoimmune glomerulonephritis showed that higher levels of MCP-1, sC5b-9, and TGF-β1 predicted the activity and adverse outcomes of the disease.^[58]^ The MCP-1/CCR2 axis plays a major role in immune cell recruitment in autoimmune nephropathy, and exhibits a potential therapeutic target for autoimmune nephropathy.

### 4.3. MCP-1/CCR2 axis and UUO nephropathy

UUO model nephropathy is a classical model of renal fibrosis characterized by progressive tubulointerstitial fibrosis. In the kidneys of UUO mice, MCP-1 expression is increased.^[59]^ In Smad3 knockout mice, inhibition of MCP-1-dependent macrophage infiltration may be a mechanism to alleviate renal fibrosis in UUO mice.^[60]^ MCP-1/CCR2 axis may be one of the main pathways of Twist1-mediated renal fibrosis in UUO mice.^[39]^ Infiltration of CCR2-positive macrophages was decreased in UUO mice, in which the N-terminus of MCP-1 was deleted.^[61]^

### 4.4. MCP-1/CCR2 axis and other renal fibrotic diseases

In addition to DN, autoimmune nephropathy, and UUO nephropathy, the MCP-1/CCR2 axis is relate to other renal fibrotic diseases. Wilkening et al induced a model of doxorubicin nephropathy in CCR2-deficient and BALB/c wild-type mice and found that CCR2-deficient mice had decreased infiltration of macrophages and fibroblasts together with inflammatory responses. Compared with wild-type mice, CCR2-deficient mice showed significant improvement in renal fibrosis, indicating that the MCP-1/CCR2 axis mediated the progression of renal fibrosis in the doxorubicin-induced nephropathy model.^[49]^ Kashyap et al performed renal artery stenosis surgery on wild-type and MCP-1 knockout mice to form a mouse model of renal vascular hypertension. They conducted a 4-week study on the mice, and found that MCP-1 knockout mice had fewer inflammatory cells and inflammatory factors than wild-type mice. In addition, fibrosis was reduced, suggesting that under the same circumstances of renal injury, kidney inflammation and fibrosis were alleviated in MCP-1 knockout mice.^[62]^ Sakata et al also showed that high sodium chloride intake could downregulate the expression of MCP-1 in 5/6 nephrectomized mice, recruit macrophages to the damaged kidney, and aggravate the progression of renal fibrosis.^[38]^ The studies indicate that inflammation plays a key role in renal fibrosis. MCP-1/CCR2 axis is an important pathway for chemotactic recruitment of inflammatory cells, and may become a promising therapeutic target for renal fibrosis.

## 5. MCP-1/CCR2 axis-associated targeted therapy of renal fibrosis

As mentioned above, the MCP-1/CCR2 axis is important in renal fibrosis, and accumulated experimental evidence provides a good foundation for clinical research. In addition, the blockade of MCP-1/CCR2 axis has been widely investigated and represents a promising therapeutic target in preclinical research and clinical trials (Table 1). Various therapeutic strategies have been designed to inhibit the activation and transduction of the MCP-1/CCR2 axis using chemokine antagonists or gene blocking.

**Table 1:** Clinical/preclinical studies on MCP-1/CCR2 axis targeting.

Targeting drug	Target	Experimental type	Experimental object/model	Method	Result
mNOX-E36-3’PEG	MCP-1	Preclinical study	db/db mice with advanced glomerulopathy	50 mg/kg, subcutaneous injection, 3 times per wk, continuous intervention for 8 wk	The number of glomerular macrophages↓, renal MCP-1 mRNA and protein ↓
RS102895	CCR2	Preclinical study	U-IRI mice	5mg/kg, intraperitoneal injection, every 12 h, continuous intervention for 7 d	Macrophages↓, dendritic cells↓, T cell↓, profibrotic growth factors↓, proimflammatory cytokines↓, extracellular matrix↓,kidney injury markers↓
RS504393	CCR2,CCR1	Preclinical study	UUO mice	2 mg/kg orally twice a day, continuous intervention time from 3 d before ureteral ligation until the day of sacrifice	MCP-1↓, TGF-β↓, collagen type I↓, F4/80-positive macrophages↓, renal interstitial fibrosis↓
Emapticap pegol (NOX-E36)	MCP-1	Clinical trial	Diabetes patients with Albuminuria	0.5 mg/kg, subcutaneous injection, twice a wk, continuous intervention for 12 wk	Urinary albumin/creatinine ratio (UACR) ↓(−29%), HbA1c↓(−0.35%)
CCX140-B	CCR2	Clinical trial	Diabetes patients with Albuminuria ([UACR] 100–3000 mg/g)	5 mg or 10 mg, once a day. continuous intervention for 52 wk	Urinary albumin/creatinine ratio (UACR) ↓, −18% for 5 mg CCX140-B, −11% for 10 mg CCX140-B

CCR2 = C–C chemokine receptor type 2, MCP-1 = monocyte chemoattractant protein-1, TGF-β = transforming growth factor-β, U-IRI = unilateral renal ischemia/reperfusion injury, UUO = unilateral ureteral obstruction.

### 5.1. Preclinical studies of targeting MCP-1/CCR2 axis in renal fibrosis

In several preclinical studies, MCP-1 and CCR2 antagonists, such as mNOX-E36-3’PEG, RS102895, and RS504393, have been successfully used to block the MCP-1/CCR2 axis conduction in treating renal fibrosis.

mNOX-E36-3’PEG, an MCP-1 antagonist, can reduce macrophage recruitment to the glomerulus and renal interstitium of diabetic mice by targeting the MCP-1/CCR2 axis and alleviating renal fibrosis in mice with experimental DN.^[63]^

RS102895 is a potent CCR2 antagonist with no effect on CCR1. Xu et al blocked CCR2 in unilateral renal ischemia/reperfusion injury (U-IRI) mice by genetic engineering or the drug RS102895 and found a decrease in macrophages, T cells, and dendritic cells in damaged kidneys and reduced fibrosis and inflammation.^[28]^ Similarly, the CCR2 antagonist RS102895 can improve renal fibrosis in U-IRI mice and IgA nephropathy.^[45,64]^

RS504393 is a selective CCR2 antagonist that acts on both the CCR2 and CCR1 receptors. RS504393 delays renal fibrosis by inhibiting the chemotaxis of CD11bLy6C monocytes in UUO-induced renal interstitial fibrosis mice. Kitagawa et al showed that CCR2-positive cells infiltrating renal interstitial fibrosis were mainly F4/80-positive macrophages. Wild-type UUO mice treated with RS504393 showed reduced MCP-1 protein, TGF-β, collagen type I, and F4/80-positive macrophages, together with renal interstitial fibrosis. There was no significant difference between the UUO mice targeted by CCR2 and wild-type UUO mice treated with RS504393. Compared with wild-type mice, renal interstitial fibrosis was alleviated in mice with CCR2 blocking or gene targeting. That indicates RS504393 can reduce inflammation and renal fibrosis by targeting MCP-1/CCR2 axis.^[65]^

In addition to directly blocking of MCP-1/CCR2 axis, indirect blocking of MCP-1/CCR2 axis can also effectively protect fibrotic kidneys. L1-10 is a specific Angpt2 inhibitor. Because the abnormal increase of Angpt2 expression promotes the migration of endothelial MCP-1 activated macrophages and cell death in renal fibrosis, L1-10 can indirectly reduce the expression of MCP-1 by inhibiting Angpt2 to protect the kidney.^[66]^

### 5.2. Clinical trials of targeting MCP-1/CCR2 axis in treating renal fibrosis

Emapticap pegol (NOX-E36) specifically binds to and inhibits MCP-1. It blocks the activation and conduction of MCP-1/CCR2 axis. Previous studies have shown that urinary protein and elevated glycosylated hemoglobin are the determining factors that promote the progression of renal fibrosis in DN. A phase II clinical trial showed that NOX-E36 was effective in controlling urinary protein and glycosylated hemoglobin levels in patients with DN. By inhibiting the MCP-1/CCR2 axis, NOX-E36 could reduce monocyte recruitment and macrophage infiltration, thereby decreasing monocyte-macrophage-induced matrix deposition and delaying renal fibrosis in DN. And the strategy was safe and well tolerated.^[67]^

CCX140-B is a CCR2 antagonist. A clinical trial involving 78 research centers showed that treatment with CCX140-B, in addition to standard treatment for type 2 diabetes and kidney disease, could further reduce urinary protein levels and protect damaged kidneys.^[68]^

## 6. Discussion

To summarize, the MCP-1/CCR2 axis is very important in renal fibrosis. It mediates and promotes renal fibrosis through chemotaxis, recruitment, activation and transdifferentiation of macrophages. The MCP-1/CCR2 axis is a potential therapeutic target for renal fibrosis. Clinical trials targeting MCP-1/CCR2 axis have shown promising results. Saito et al showed MCP-1 and CCR2 levels were positively correlated with renal fibrosis. However, inhibition of the MCP-1/CCR2 axis with drugs could downregulate CCR2, but it was not sufficient to prevent acute kidney injury from progressing to CKD or to improve renal fibrosis in mice with U-IRI.^[69]^ Similarly, Salah et al performed MCP-1 genetic knockout in mice with congenital polycystic kidneys to test whether deletion of MCP-1 reduced renal macrophage infiltration and slowed disease progression. However, the results were surprising: there was no significant reduction in renal macrophages and no improvement in cystic lesions or renal function, but genetic knockout of MCP-1 prevented pulmonary edema in mice with congenital polycystic kidneys and increased survival in mice.^[70]^ The results of inhibition of MCP-1/CCR2 axis in treating renal fibrosis are not completely consistent, and the reasons for this are still unclear. The results may be different depending on the experimental designs, timing of drug interventions, and animal models. Moreover, there are other signal pathways involved in renal fibrosis, and the biological function of MCP-1/CCR2 axis is regulated by various factors. For example, TGF-β1 is a critical mediator of renal fibrosis, and it can upregulate MCP-1 in rat renal tubular epithelial cells.^[71]^ Further study of cross-talk between TGF-β1 and MCP-1/CCR2 may deepen the understanding of the pathogenesis of renal fibrosis. Moreover, preventing the production of MCP-1 stimulated by TGF-β1 may be a promising therapeutic strategy for delaying renal fibrosis. In addition, MCP-1/CCR2 axis may also have a protective effect on renal fibrosis. MCP-1 is released from renal tubular epithelial cells after renal injury, which promotes CCR2 positive monocyte to flow into the injured site, and monocyte differentiate into M1 or M2 macrophages. M1 macrophages can produce pro-inflammatory cytokines such as TNF-α, IL-1β, and IL-6, promoting the progression of renal fibrosis. M2 macrophages can produce IL-10, which participates in the repair of renal fibrosis tissues.^[2]^ After the MCP-1/CCR2 axis is activated, the recruited CCR2 positive monocyte differentiate into M1 or M2 macrophages, the type of which may depend on the stage of renal fibrosis. The MCP-1/CCR2 axis may be harmful or beneficial at different stages of renal fibrosis. It is particularly important to identify the different stages of renal fibrosis and select the timing of targeted therapy, which poses a greater challenge to the targeted MCP-1/CCR2 axis in the treatment of renal fibrosis.

## 7. Conclusions

Renal fibrosis mediated by MCP-1-CCR2 axis involves a series of cellular or molecular signaling pathways and the recruitment of various immunosuppressive cells (especially monocyte macrophage cells). It is difficult to achieve significant clinical benefits in the treatment of renal fibrosis by targeting MCP-1 or CCR2 alone. Therefore, more attention should be paid to comprehensively investigating the underlying mechanisms of MCP-1/CCR2 axis in renal fibrosis. MCP-1/CCR2 axis seems to play a bridging role in the inflammatory cascade of renal fibrosis. An in-depth study of the role of MCP-1/CCR2 axis in the inflammatory response and complex immune cell signaling network may provide new ideas for treating renal fibrosis, benefiting an increasing number of patients with CKD.

## Author contributions

**Conceptualization:** Shiyang He.**Resources:** Lan Yao.

**Supervision:** Lan Yao.

**Writing – original draft:** Shiyang He.

**Writing – review & editing:** Jun Li.
